# Identification of fibroblast progenitors in the developing mouse thymus

**DOI:** 10.1242/dev.200513

**Published:** 2022-05-26

**Authors:** Pedro Ferreirinha, Ruben G. R. Pinheiro, Jonathan J. M. Landry, Nuno L. Alves

**Affiliations:** 1 Instituto de Investigação e Inovação em Saúde, Universidade do Porto, 4200-135, Porto, Portugal; 2 Instituto de Biologia Molecular e Celular, 4200-135, Porto, Portugal; 3 Doctoral Program in Molecular and Cell Biology, Instituto de Ciências Biomédicas Abel Salazar, Universidade do Porto, 4200-135, Porto, Portugal; 4 Genomics Core Facility, European Molecular Biology Laboratory, 69117 Heidelberg, Germany

**Keywords:** Thymic mesenchymal cells, Thymic stroma, Thymus, Progenitors, Mouse

## Abstract

The thymus stroma constitutes a fundamental microenvironment for T-cell generation. Despite the chief contribution of thymic epithelial cells, recent studies emphasize the regulatory role of mesenchymal cells in thymic function. Mesenchymal progenitors are suggested to exist in the postnatal thymus; nonetheless, an understanding of their nature and the mechanism controlling their homeostasis *in vivo* remains elusive. We resolved two new thymic fibroblast subsets with distinct developmental features. Whereas CD140αβ^+^GP38^+^SCA-1^−^ cells prevailed in the embryonic thymus and declined thereafter, CD140αβ^+^GP38^+^SCA-1^+^ cells emerged in the late embryonic period and predominated in postnatal life. The fibroblastic-associated transcriptional programme was upregulated in CD140αβ^+^GP38^+^SCA-1^+^ cells, suggesting that they represent a mature subset. Lineage analysis showed that CD140αβ^+^GP38^+^SCA-1^+^ maintained their phenotype in thymic organoids. Strikingly, CD140αβ^+^GP38^+^SCA-1^−^ generated CD140αβ^+^GP38^+^SCA-1^+^, inferring that this subset harboured progenitor cell activity. Moreover, the abundance of CD140αβ^+^GP38^+^SCA-1^+^ fibroblasts was gradually reduced in *Rag2*^−/−^ and *Rag2*^−/−^*Il2rg*^−/−^ thymi, indicating that fibroblast maturation depends on thymic crosstalk. Our findings identify CD140αβ^+^GP38^+^SCA-1^−^ as a source of fibroblast progenitors and define SCA-1 as a marker for developmental stages of thymic fibroblast differentiation.

## Abstract (precis)

**Summary:** This study resolves previously unidentified subsets of immature and mature thymic fibroblasts, providing further evidence that their homeostasis is controlled by signals provided by developing thymocytes.

## INTRODUCTION

The thymic microenvironment offers a unique inductive site for the generation of functionally diverse and self-tolerant T cells. The thymic stroma is formed by cells of non-haematopoietic origin, such as thymic epithelial cells (TECs), endothelial cells and thymic mesenchymal cells (TMCs), and cells of haematopoietic origin, including dendritic cells and monocytes/macrophages (James et al., 2021a). The development of this heterogeneous microenvironment starts in the embryo and continues during postnatal life, involving the participation of cells from all three embryonic germ layers: endoderm-derived epithelium, neuroectoderm-derived neural-crest (NC) mesenchyme and mesoderm-derived haematopoietic and endothelial cells (Gordon and Manley, 2011). Given the non-redundant role of TECs in T-cell development, there has been considerable interest in studying the mechanisms that control TEC differentiation and function. However, several studies underscore the contribution of other non-epithelial stromal cells in shaping TEC and T-cell differentiation (Nitta et al., 2021).

In particular, TMCs, including fibroblasts, vascular-supporting pericytes and smooth muscle cells, exert a pleiotropic role in thymus biology (Nitta et al., 2021). At an early stage of thymus organogenesis, NC-derived mesenchymal cells surround the thymic primordia and provide fibroblast growth factor 7 (FGF7), FGF10, epidermal growth factor (EGF) and insulin-like growth factor (IGF), which contribute to the growth of the TEC microenvironment (Jenkinson et al., 2003; Jenkinson et al., 2007). Interestingly, FGF7/10-producing cells also express retinoic acid, which suppresses the proliferation of cortical TECs (Sitnik et al., 2012; Wendland et al., 2018). Thus, TMCs have the functional capacity to positively and negatively control the size of the TEC compartment. Thymic fibroblasts also produce a range of extracellular matrix (ECM) components, which can capture and present crucial thymopoietic factors (e.g. IL7 and CCL21) to the developing T cells (Banwell et al., 2000; James et al., 2021b). Moreover, vascular-associated pericytes and smooth muscle cells surrounding the endothelium regulate thymic vasculature and T-cell egress (Zachariah and Cyster, 2010; Sitnik et al., 2016). Particularly, TMCs create sphingosine-1-phosphate (S1P) gradients that promote the egress of mature T cells from the thymus (Zachariah and Cyster, 2010). More recently, medullary fibroblasts have been implicated in T-cell tolerance (Nitta et al., 2020). Despite the aforementioned functional diversity, distinct TMC subsets share a precursor-product relationship with NC cells (Müller et al., 2008; Foster et al., 2008; Sitnik et al., 2016). Still, our understanding of the mechanisms that control the differentiation and the turnover of mature TMCs remains incomplete. Moreover, although thymic mesenchymal progenitors are considered to exist in the adult thymus (Sitnik et al., 2016), their nature and functional competence remain poorly characterized *in vivo*.

Herein, we resolved a previously unidentified population of thymic fibroblast progenitors and uncovered a checkpoint in mesenchymal differentiation that depends on thymic crosstalk. Our findings offer a roadmap to monitor TMC homeostasis in ageing and regeneration.

## RESULTS AND DISCUSSION

### Analysis of thymic fibroblast differentiation during development

**Fig. 1.**
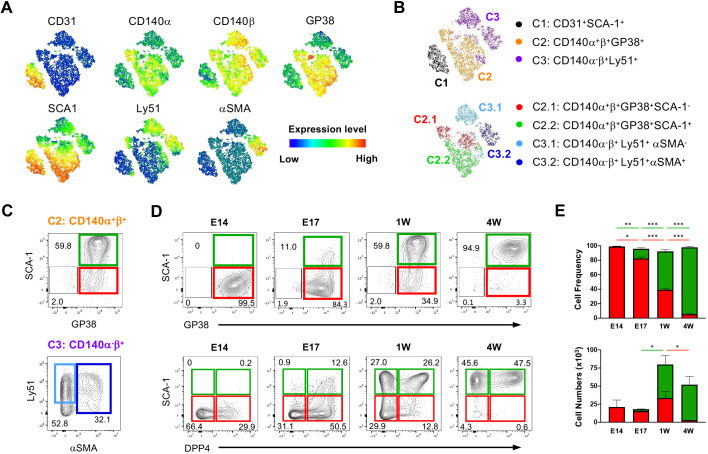
**GP38 and SCA1 expression on TMC subsets.** (A) Total thymi cells from 1-week-old mice were isolated, and total TMCs (CD45^−^EpCAM^−^) were analysed by flow cytometry. t-SNE representation of the expression of CD31, CD140α, CD140β, GP38, SCA-1, Ly51 and αSMA. (B) Three main clusters were identified: cluster 1 (CD31^+^), cluster 2 (CD140α^+^β^+^) and cluster 3 (CD140α^−^β^+^). Clusters 2 and 3 were respectively subdivided into cluster 2.1 (CD140α^+^β^+^GP38^+^SCA1^−^) and 2.2 (CD140α^+^β^+^GP38^+^SCA1^+^); and 3.1 (CD140α^−^β^+^Ly51^+^αSMA^−^) and 3.2 (CD140α^−^β^+^Ly51^+^αSMA^+^). (C) TMCs (CD45^−^EpCAM^−^CD31^−^) were analysed for the indicated markers, and sub-cluster 2.1 (red gate), 2.2 (green gate), 3.1 and 3.2 (light- and dark-blue gates) were identified. (D) Analysis of GP38, SCA-1 and DPP4 expression in TF^A^ (red gate) and TF^B^ (green gate) populations at E14, E17, 1 week old (W) and 4W. Numbers in plots indicate the frequency of cells found within each gate. Plots are of a representative analysis per time point. (E) Bar graphs showing mean+s.d. of the frequency and cellularity of TF^A^ and TF^B^ subsets, of three independent analyses per time point. Differences in TF subsets, CD140α^+^β^+^GP38^+^SCA1^−^ (red) and CD140α^+^β^+^GP38^+^SCA1^+^ (green), were statistically analysed at different ages: ***P<0.001, **P<0.01, *P<0.05.

Several markers, including CD140α (PDGFRA), CD140β (PDGFRB), GP38 (PDPN), ER-TR7, MTS-15, SCA-1 (Ly6a), αSMA (ACTA2), CD146 (MCAM), CD34, Ly51 (ENPEP), Itga7 and DPP4 have been used to phenotypically identify specific populations of TMCs (Jenkinson et al., 2003; Jenkinson et al., 2007; Gray et al., 2007; Foster et al., 2008; Sitnik et al., 2012; Patenaude and Perreault, 2016; Sitnik et al., 2016; Sheridan et al., 2017; Nitta et al., 2020). Nonetheless, as some of these markers are also expressed by other cell types, they cannot specifically define distinct differentiation states of TMCs when employed in a restrictive manner. To dissect the heterogeneity within TMCs, we sought to identify cells expressing progenitor hallmarks within the entire postnatal mesenchymal compartment. We selected the postnatal day 7 thymus, as a period when the main haematopoietic, epithelial and mesenchymal subsets were present. Employing multiparameter flow cytometry, we analysed the expression of ten well-known cell-surface markers. To discriminate haematopoietic, epithelial, endothelial and erythroid lineages, we included CD45 (PTPRC), EpCAM, CD31 (PECAM1) and Ter119 (Ly76), respectively. For the analysis of TMCs, we initially considered the following markers: CD140α, CD140β, GP38, SCA-1, Ly51 and αSMA. Flow cytometry data of non-haematopoietic and non-epithelial cells was analysed by nonlinear dimensionality reduction algorithms, producing maps that clustered cells based on their phenotypic similarity [t-distributed stochastic neighbour embedding (t-SNE)] (Fig. 1A). This unsupervised approach revealed three main clusters within CD45^−^EpCAM^−^ cells. Cluster 1 was formed by CD31^+^SCA-1^+^ cells, cluster 2 comprised CD140α^+^β^+^GP38^+^ cells, and cluster 3 contained CD140α^−^β^+^Ly51^+^ cells (Fig. 1B). Changes in SCA-1 and αSMA expression, respectively, showed an additional layer of heterogeneity within clusters 2 and 3: whereas the differential expression of SCA-1 identified sub-clusters 2.1 (CD140α^+^β^+^GP38^+^SCA-1^−^) and 2.2 (CD140α^+^β^+^GP38^+^SCA-1^+^), alterations in αSMA expression distinguished sub-clusters 3.1 (CD140α^−^β^+^Ly51^+^αSMA^−^) and 3.2 (CD140α^−^β^+^Ly51^+^αSMA^+^) (Fig. 1B). Employing a directed gating strategy, we identified the same TMC subsets: CD140α^+^β^+^GP38^+^SCA-1^−^ (2.1), CD140α^+^β^+^GP38^+^SCA-1^+^ (2.2), CD140α^−^β^+^Ly51^+^αSMA^−^ (3.1) and CD140α^−^β^+^Ly51^+^αSMA^+^ (3.2) (Fig. 1C, Fig. S1). These results suggested that cluster 1 defined endothelial cells, cluster 2 included fibroblasts and cluster 3 identified endothelial-supporting mesenchymal cells, which can be further subdivided into pericytes (3.1) and smooth muscle cells (3.2) (Sitnik et al., 2016). Our observations further showed that the differential expression of CD140α can be used to distinguish fibroblasts (CD140α^+^β^+^) from pericyte-like cells (CD140α^−^β^+^). Moreover, SCA-1-expressing thymic fibroblasts (2.2) have been previously reported (Patenaude and Perreault, 2016; Sheridan et al., 2017). Yet, the segregation of CD140α^+^β^+^GP38^+^ in SCA-1^−^ (2.1) and SCA-1^+^ (2.2) was intriguing and led us to direct our attention to these subsets. We refer hereafter to cells within cluster 2.1 (CD140α^+^β^+^GP38^+^SCA-1^−^) and cluster 2.2 (CD140α^+^β^+^GP38^+^SCA-1^+^) as thymic fibroblast A (TF^A^) and B (TF^B^), respectively.

To examine whether TF^A^ and TF^B^ defined two distinct subsets, we analysed their development during thymic ontogeny and postnatal life. TF^A^ predominated at embryonic day (E) 14 and their numbers were relatively constant up to the first week of postnatal life, followed by a decrease in the 4-week-old thymus. Contrarily, TF^B^ cells arose around E17 and expanded in frequency and number during the perinatal period (E17 to 4 weeks old) (Fig. 1D,E). We further addressed how the differentiation of TF^A^ and TF^B^ related to recently described medullary (DPP4^−^) and capsular (DPP4^+^) fibroblasts (Nitta et al., 2020). At E14.5, a period wherein TF^B^ were virtually absent, TF^A^ contained DPP4^+^ and DPP4^−^ cells. The first TF^B^ (SCA-1^+^) appeared at E17 and were mostly DPP4^+^, suggesting that their immediate precursors could be within the TF^A^DPP4^+^ population. From the postnatal period onwards, TF^B^ contained both DPP4^−^ and DPP4^+^ cells (Fig. 1D). A population of TF^A^ expressing low levels of DPP4 persisted in 1-week-old thymi (Fig. 1D). In line with a previous report (Nitta et al., 2020), the observation that DPP4^−^ and DPP4^+^ cells appeared in the early embryonic TF^A^ subset may suggest that segregation of capsular and medullary sub-lineages occurs early in thymic development. Moreover, our results indicate that SCA-1 expression was acquired firstly by capsular (DPP4^+^) fibroblast followed by medullary (DPP4^−^) counterparts. As such, the acquisition of SCA-1 expression appears to represent a maturation marker commonly acquired by capsular and medullary thymic fibroblasts and does not by itself discriminate these subsets. The developmental kinetic of TF^A^ and TF^B^ led us to consider that they could represent distinct stages of the same differentiation pathway. In this scenario, TF^A^ should contain precursors with the potential to differentiate into TF^B^. Alternatively, TF^A^ and TF^B^ could define unrelated thymic mesenchymal cells. We conducted genome-wide transcriptional and lineage-tracing experiments to investigate further the precursor-product relationship between these subsets.

### TF^A^ and TF^B^ subsets have distinct transcriptional programmes

**Fig. 2.**
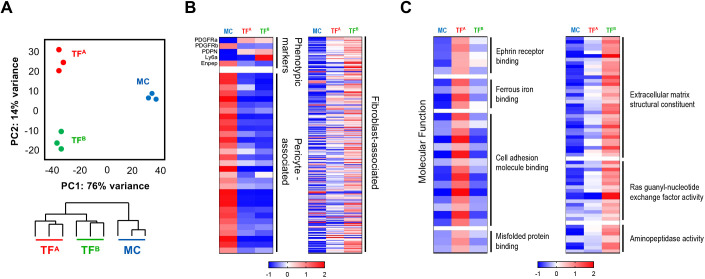
**Genome-wide transcriptomic analysis of TF subsets identifies stages with distinctive gene expression profiles.** (A) Principal component analysis plot and dendrogram, detailing the hierarchical clustering between the biological samples, performed with data obtained from total RNA-sequencing analysis of sorted TF^A^ (CD45^−^EpCAM^−^GP38^+^SCA-1^−^) (*n*=3), TF^B^ (CD45^−^EpCAM^−^GP38^+^SCA-1^+^) (*n*=3) and MC (CD45^−^EpCAM^−^GP38^−^SCA-1^−^Ly51^+^) (*n*=3) populations. (B) Heat maps representing the deviation from average expression of the phenotypic markers used to identify TMC populations, of genes previously associated with pericytes and of genes previously associated with thymic fibroblasts. (C) Heat maps representing the deviation from average expression of the uniquely upregulated genes identified for populations TF^A^ and TF^B^ and the associated molecular functions identified by GO analysis. Genes with FDR<10% were considered as differentially expressed. Enriched GO terms (molecular functions) were identified using MGSA. Represented categories had a marginal posterior probability estimate higher than 0.65.

To examine whether TF^A^ and TF^B^ identified different states of fibroblast differentiation, we characterized their genome-wide transcriptional profile by employing RNA-sequencing analysis. TF^A^ and TF^B^ were purified by cell sorting from the 1-week-old thymus, a period wherein these subsets were equally represented. Additionally, we purified endothelial-supporting mural cells (MCs) (cluster 3) and included them as a complementary reference population in the transcriptional analysis. Principal component analysis showed that the biological replicates of each subset clustered together, demonstrating that these populations had low intrapopulation variability. Moreover, TF^A^ and TF^B^ were more closely related to each other than to MCs (Fig. 2A, Fig. S2A, Table S1). Employing available transcriptomic data sets from other studies (Patenaude and Perreault, 2016; Sitnik et al., 2016; Nitta et al., 2020), we extracted sets of genes associated with fibroblasts, vascular-supporting cells, and cross-examined their expression pattern in TMC subsets. First, the expression of genes used as phenotypic markers to define TF^A^, TF^B^ and MC subsets followed the expected pattern, validating the accuracy of the purified samples. Second, most fibroblasts-associated genes were upregulated in TF^A^ to TF^B^, whereas transcripts linked to vascular-supporting cells were specifically enriched in MCs (Fig. 2B, Table S2). Moreover, an unsupervised cross-analysis of genes linked to capsular and medullary fibroblasts (Nitta et al., 2020), revealed that these transcripts were greatly increased in TF^B^ (Fig. S2B, Tables S3, S4). These observations were in line with the representation of capsular and medullary subsets within TF^A^ and TF^B^ in the 1-week-old thymus (Fig. 1E) and support their fibroblastic identity. Further bioinformatic analysis identified 470 and 721 uniquely upregulated genes in TF^A^ and TF^B^, respectively (Fig. S2C, Tables S5, S6). Gene ontology (GO) enrichment analysis of these sub-lineage specific sets revealed a stringent association to diverse functional categories. Specifically, genes enriched in TF^A^ were linked to broad cellular processes, including ephrin receptor signalling, cell adhesion, binding to iron and misfolded protein. By contrast, genes upregulated in TF^B^ were associated with more restricted processes, including ECM components, GTPase signalling and aminopeptidase activity (Fig. 2C, Tables S7, S8). Several collagen genes were upregulated in TF^B^, consistent with the association with ECM constituents (Fig. S2D, Table S9). Recent findings implicated LTβR-mediated signalling in thymic medullary fibroblast differentiation (James et al., 2018; Nitta et al., 2020). Detailed analysis of members of the TNFRSF family showed that *Ltbr*, *Tnfrsf1b*, *Tnfrsf12a* and *Tnfrsf23* were specifically upregulated in TF^B^ (Fig. S2E, Table S6). Together, our results suggest that TF^A^ may contain more immature cells, whereas TF^B^ appear to define mature thymic fibroblasts.

### TF^A^ can give rise to TF^B^ and their homeostasis is altered in the alymphoid thymus

**Fig. 3.**
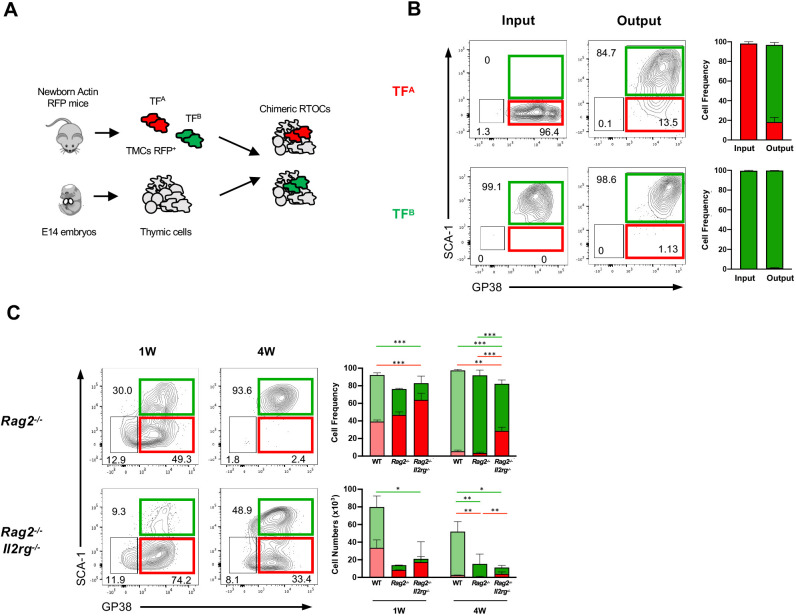
**TF^A^ contains progenitor cells capable of generating TF^B^, in a process dependent on thymic crosstalk.** (A) Chimeric RTOCs were established with E14 cells from WT thymus and mixed with TF^A^ or TF^B^ cells isolated from the postnatal day 1-3 Actin-RFP mice. (B) Flow cytometry analysis of the chimeric RTOC at day 0 (input) and after 7 days in culture (output). Data presented and bar graphs correspond to mean+s.d. of two independent analyses. (C) Analysis of GP38 and SCA-1 expression within TF populations from 1- and 4-week-old *Rag2*^−/−^ and *Rag2*^−/−^*Il2rg*^−/−^ mice. Numbers in plots indicate the frequency of cells found within each gate. Flow cytometry plots are of a representative analysis. Bar graphs correspond to mean+s.d. of two (1-week-old *Rag2*^−/−^) and three (1-week-old *Rag2*^−/−^*Il2rg*^−/−^ and 4-week-old *Rag2*^−/−^ and *Rag2*^−/−^*Il2rg*^−/−^) independent experiments per time point. Each experiment contains a pool of two to four mice per analysis. The numbers of TF subsets found in the WT thymus are co-represented as a reference and were originally described in Fig. 1. Differences between WT and *Rag2*^−/−^*Il2rg*^−/−^ TF subsets at 1 week and between WT, *Rag2*^−/−^ and *Rag2*^−/−^*Il2rg*^−/−^ at 4 weeks were statistically analysed: ***P<0.001, **P<0.01, *P<0.05.

The observations that TF^B^ developed at E17 presumably from TF^A^ suggested a possible precursor-product lineage relationship between these populations. To assess this hypothesis, we first established fetal thymic organ cultures (FTOCs) with E14 thymi, a stage at which TF^B^ were virtually absent. TF^B^ emerged after 4 days of culture, partially phenocopying the composition of TF subsets in the E17 thymus (Fig. S3A). These results suggested that TF^B^ precursors already existed in the E14 thymus and that subsequent intrathymic interactions may promote their differentiation. To determine the lineage potential of TF^A/B^ in the postnatal thymus, we purified (by fluorescence-activated cell sorting) these populations from 1-week-old-thymus and established reaggregate thymus organ cultures (RTOCs). TF subsets were isolated from the thymus of Actin^RFP^ reporter mice (Meireles et al., 2017) and mixed with wild type (WT)-derived embryonic thymic cells (carriers). In this system, RFP expression is constitutively active in ‘spiked’ cells (TF^A/B^), providing an intrinsic label for lineage-tracing analysis of TF subsets (Fig. 3A, Fig. S3B). The differentiation potential of TF subsets was analysed after 7 days of culture. Whereas TF^B^ largely maintained their phenotype, TF^A^ gave rise to TF^B^ (Fig. 3B). None of the two subsets originated vascular-supporting cells (CD140α^−^β^+^Ly51^+^) (data not shown). In both RTOCs, embryonic carrier cells (RFP^−^), which are mostly composed of TF^A^, followed the same differentiation trajectory (Fig. S3B,C). These results suggested that TF^B^ represents a more committed fibroblast population, whereas the TF^A^ population contains cells with fibroblast progenitor activity.

It is well recognized that the establishment of epithelial microenvironments depends on functional bidirectional interactions between haematopoietic cells and TECs (Rodrigues et al., 2018). A recent study showed that the differentiation of thymic medullary fibroblasts also depends on signals provided by developing thymocytes (Nitta et al., 2020). Thymic organotypic cultures allow the normal programme of T-cell and TEC differentiation (Ribeiro et al., 2013; Meireles et al., 2017). Thus, the observations that TF^A^ gave rise to TF^B^ in FTOC and RTOC led us to consider whether there was a stage-specific requirement for thymocyte crosstalk during thymic fibroblast differentiation. To evaluate this possibility, we analysed TF development in mutant mice in which thymocyte development is inhibited at different stages. Whereas in *Rag2*^−/−^ mice T-cell development is blocked at the double negative (DN) 3 stage, *Rag2*^−/−^*Il2rg*^−/−^ mice display a premature and more severe arrest in thymocyte development (Ribeiro et al., 2013; Meireles et al., 2017). Relative to the WT thymus, the proportion of TF^B^ was profoundly affected in the 1- and 4-week-old *Rag2*^−/−^*Il2rg*^−/−^ thymus, leading to an accumulation of GP38^−/low^ cells and an overall reduced GP38 expression at 1 and 4 weeks of age (Fig. 3C). The frequency of TF^B^ in *Rag2*^−/−^ thymus was also reduced in the 1-week-old-thymus relative to WT counterparts, although to a lesser extent compared with *Rag2*^−/−^*Il2rg*^−/−^. However, the representation TF^B^ in *Rag2*^−/−^ thymus at 4 weeks was similar to that observed in the WT thymus. Strikingly, the numbers of TF^B^ were markedly reduced in both 1- and 4-week-old *Rag2*^−/−^ and *Rag2*^−/−^*Il2rg*^−/−^ thymus compared with WT counterparts (Fig. 3C). The results in the *Rag2*^−/−^*Il2rg*^−/−^ thymus cannot formally exclude an additional role for γ_c_-mediated signalling in thymic fibroblast differentiation. Some reports indicate that γ_c_ cytokine family may also affect the function of non-haematopoietic stromal cells, such as endothelial cells (Leonard et al., 2019). However, the observation that TF^B^ differentiation was also impaired in the *Rag2*^−/−^ thymus, wherein γ_c_-mediated signalling was intact, supports the hypothesis that thymic fibroblast maturation is controlled by cooperative signals provided by thymocytes passing the β selection checkpoint. In this regard, the maturation of medullary fibroblast also required cellular interactions with mature TCRαβ-expressing thymocytes (Nitta et al., 2020). Moreover, it remains unknown whether mature thymic fibroblasts in the adult thymus are replaced by dedicated progenitors. A mesenchymal progenitor population referred to as CD34^+^ adventitial cells (CD34^+^GP38^+^) has been previously reported to exist in the adult thymus (Sitnik et al., 2016), and adult-derived CD34^+^ adventitial cells presented bipotent mesenchymal potential capable of generating fibroblast and pericytes (Sitnik et al., 2016). TF^A^ isolated within the postnatal thymus revealed a more fibroblastic-restricted progenitor activity. Further studies should determine whether CD34^+^ adventitial cells and TF^A^ are developmentally unrelated or define distinct stages of the same TMC differentiation process. Moreover, future analysis should resolve whether DPP4^−^ and DPP4^+^ existing within TF^A/B^ at different stages of life represent unipotent or bipotent precursors of thymic capsular and medullary fibroblasts. The decline of TF^A^ with age within the normal thymus, and their maintenance in *Rag^−/−^Il2rg*^−/−^, suggests that the pool of TF progenitors is negatively regulated by thymic crosstalk. Interestingly, a similar feedback mechanism has been reported for distinct progenitor TEC subsets. In particular, the maturation of medullary TEC depends on the cooperative role of TNFR superfamily members, including receptor activator of NF-κB (RANK), lymphotoxin β receptor (LTβR) and CD40, which are stimulated by their respective ligands expressed in several haematopoietic cells, namely lymphoid tissue inducer cells, γδ T cells, positively selected double-positive (DP) thymocytes and αβ CD4^+^ single-positive (SP4) thymocytes (Rossi et al., 2007; Hikosaka et al., 2008; Akiyama et al., 2008; Mouri et al., 2011; Desanti et al., 2012; Roberts et al., 2012). Our results suggest that cooperative signals derived from thymocytes that passed the β selection checkpoint control thymic fibroblast differentiation. These findings indicate that thymocyte-derived signals have a dual effect on thymic stromal differentiation, promoting the differentiation of mature lineage while depleting the bioavailability of the pool of distinct progenitor cells. Further studies are required to elucidate the signals that control the turnover of thymic fibroblasts *in vivo* and whether this process entails direct thymocyte-fibroblast interactions or is mediated by other cell-cell contacts.

In summary, our study resolves the identity of previously unidentified populations of thymic fibroblast precursors and exposes a checkpoint in TF differentiation that is controlled by thymic crosstalk *in vivo*. These findings represent a roadmap to understanding the processes underlying the establishment of thymic mesenchymal cells in regular and deficient thymopoiesis.

## MATERIALS AND METHODS

### Mice

WT, *Rag2*^−/−^, *Rag2*^−/−^*Il2rg*^−/−^ and Actin-RFP mice (Ribeiro et al., 2013; Ribeiro et al., 2014) were all bred on a C57BL/6 background and housed under specific pathogen-free conditions at the I3S animal facility. Experiments were performed under the European guidelines for animal experimentation.

### Isolation of thymic stromal cells

Thymic stromal cells were isolated using a protocol previously described to obtain TECs (Meireles et al., 2017), with modifications. Briefly, the thymus was cut into small pieces and subjected to a gentle mechanical dissociation to liberate thymocytes. Thymic fragments were digested for 30 min at 37°C with agitation in PBS containing 20 mg/ml of collagenase D (Roche) and passed through 100-µm filter to remove debris. Further stromal cell enrichment was carried out by incubation with anti-CD45 microbeads (Miltenyi Biotec) according to the manufacturer's instructions.

### Flow cytometry

TMCs were isolated as described (Meireles et al., 2017). Cell suspensions were stained with the following antibodies: PerCP-Cy5-conjugated anti-CD45.2 (clone 104, 45-0454-82), PE-conjugated anti-Ly51 (clone 6C3, 12-5891-82), Alexa eFluor 647-conjugated anti-EpCAM (clone G8.8, 14-5791-81), APC-conjugated anti-Ter-119 (clone TER-119, 17-5921-82), all from eBioscience; BV421-conjugated anti-EpCAM (clone G8.8, 118225), BV786-conjugated anti-Sca1 (clone D7, 108139), Alexa 488-conjugated anti-Sca1 (clone D7, 108111), PE-Cy7-conjugated anti-GP38 (clone 8.1.1, 127411), APC-conjugated anti-DPP4 (clone H194-112, 137807), BV605-conjugated anti-CD140α (clone APA5, 135916), all from BioLegend; biotinylated anti-CD140β (clone APB5, 136009, BioLegend) was revealed with BV711-conjugated (405241, BioLegend) or PE-Cy7-conjugated streptavidin (SA1012, eBioscience). Intracellular staining with eFluor 660-conjugated anti-αSMA (clone 1A4, 50-9760-82, eBioscience) was performed following cell fixation and permeabilization using the Foxp3/Transcription factor staining buffer set (eBioscience) according to the manufacturer's instructions. Flow cytometry analyses were performed on a LSRFortessa and cells sorted on a FACS ARIA II (both from BD Bioscience) with purities above 95%. Data were analysed using FlowJo software (Tree Star Inc).

### RNA sequencing

Total RNA library preparation and high-throughput sequencing of sorted postnatal (P3-5) TF^A/B^ and MC subsets were performed at the EMBL Genomics Core facility (Germany), as previously described (23). Nine sequencing libraries, three for TF^A^, three for TF^B^ and three for MCs, were prepared using NEB Next RNA ultra protocol (E7530 NEB). Obtained libraries were quantified fluorometrically, pooled in equimolar amounts and sequenced on an Illumina NextSeq 500 sequencer in single-end mode (75 bases), following the manufacturer's instructions (Illumina). The reads were mapped to the mouse genome (GRCm38) using STAR (version 2.4.2a) with GRCm38.99 GTF annotation. The number of reads per gene was generated during the alignment step (quantMode GeneCounts) and gene counts were then analysed with the DESeq2 package (24). Genes with FDR <10% were considered as differentially expressed. Enriched GO terms (biological processes and molecular functions) for the differentially expressed genes were identified using model-based gene set analysis (MGSA) (Bauer et al., 2010). The analysis was performed with ten independent runs of the Markov chain of 1.10^8^ steps each. The parameters p, alpha and beta were used as default. Functional categories with a marginal posterior probability estimate higher than 0.65 were retained for further analysis. The hierarchical clustering, represented as a dendrogram, of TEC populations was performed using the hclust function in R on Euclidean distances between the variance of the rlog-transformed read counts for each gene across samples.

### FTOCs

FTOCs were established as previously described (Ribeiro et al., 2013; Meireles et al., 2017) by placing isolated thymic lobes obtained from E14 C57BL/6 embryos on a 0.8 mm Isopore membrane filter (Millipore, ATTP01300) over a submerged foam sponge in DMEM medium supplemented with 10% FCS, 1% L-glutamine 200mM (Gibco). On the indicated days, FTOCs were dissociated and analysed by flow cytometry as previously described.

### RTOCs

RTOCs were established as previously described (Ribeiro et al., 2013; Meireles et al., 2017) by combining 7×10^5^ total thymic cells obtained from WT C57BL/6 thymus and 3.5-4×10^4^ sorted TF^A/B^ subsets obtained from newborn Actin-RFP C57BL/6 thymic lobes. After 7 days in culture, RTOCs were dissociated and analysed by flow cytometry as previously described.

### Statistical analysis

Statistical analyses were performed using GraphPad software, Version 9. Column graphs show mean+s.d. Statistical analysis was performed using two-tailed *t*-tests.

## Supplementary Material

Supplementary information

Reviewer comments
