# Supplementary material for: Identification of fibroblast progenitors in the developing mouse thymus
Source: Development. 2022 May 26;149(10):dev200513. doi: 10.1242/dev.200513 (PMC9188757; doi:10.1242/dev.200513)
Supplement: Supplementary information [file develop-149-200513-s1.pdf]

Supplementary Figure 1

A

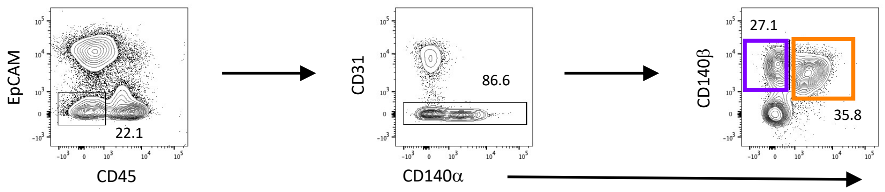

Fig. S1. Gating strategy for flow cytometry analysis of thymic mesenchymal cells. (A) Representative analysis of cells obtained from 1 week-old thymus depicting the gating strategy used to identify TMCs defined as clusters 2 and cluster 3. Numbers in plots indicate the frequency of cells found within each gate.

Development • Supplementary information

Supplementary Figure 2

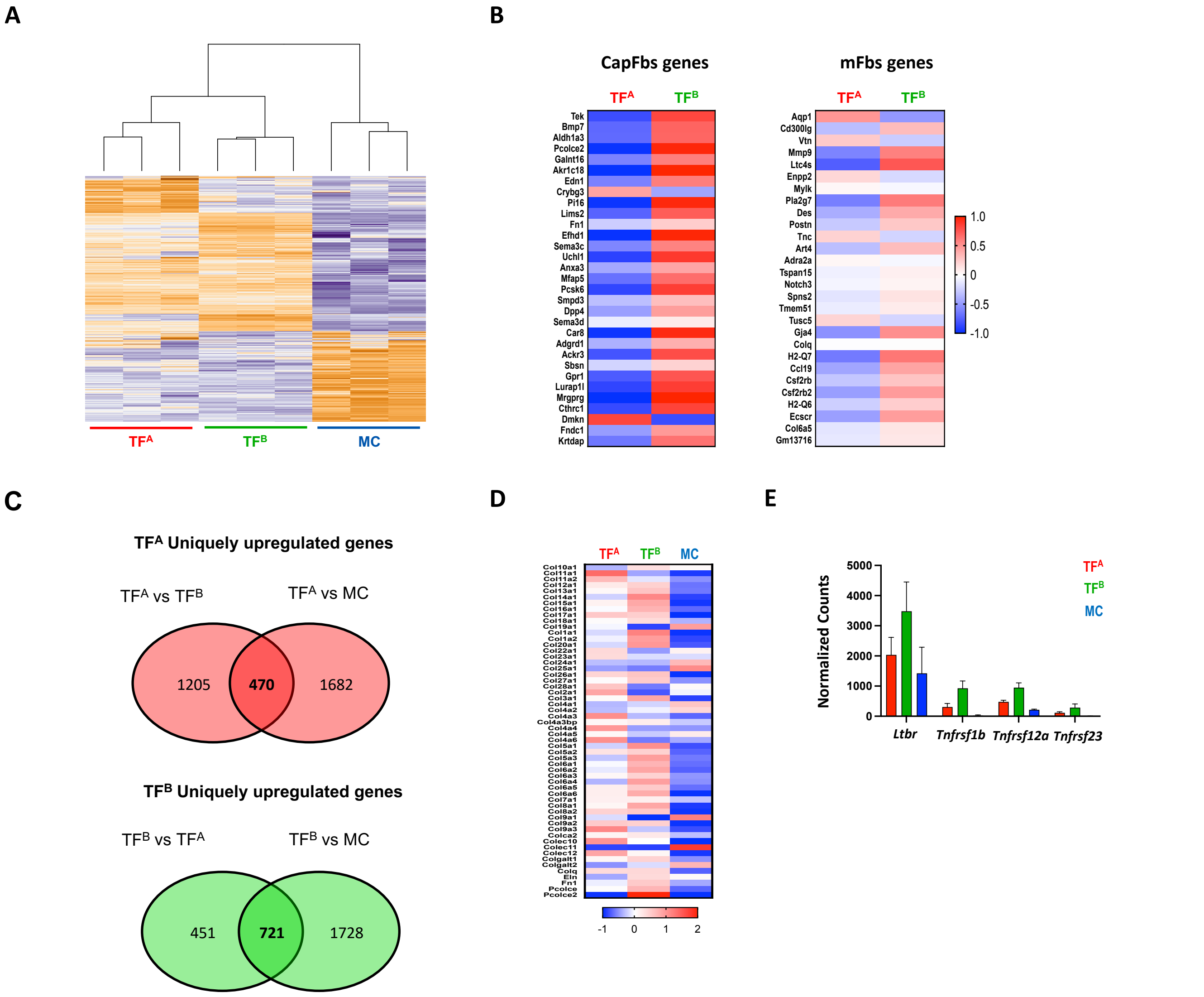

Fig. S2. RNA sequencing analysis of TMC subsets. (A) Heat map representing the 1000 most expressed genes in the assessed TMC populations and associated dendrogram detailing the hierarchical clustering between the biological samples. **(B)** Heat maps represent the deviation from the average expression of the top expressed genes associated with capsular and medullary fibroblasts as described in (Meireles et al., 2017). **(C)** Venn diagrams represent the identification of the 470 and 721 uniquely upregulated genes in TF^{A} (red) and TF^{B} (green) populations, respectively. Genes with FDR < 10% were considered as differentially expressed. **(D)** Heat maps represent the deviation from the average expression of the different collagen and collagen associated genes in the different TMC populations. **(E)** Bar graph representing the mean plus SD expression value of the TNFRSF family genes upregulated in TF^{B}.

Development • Supplementary information

Supplementary Figure 3

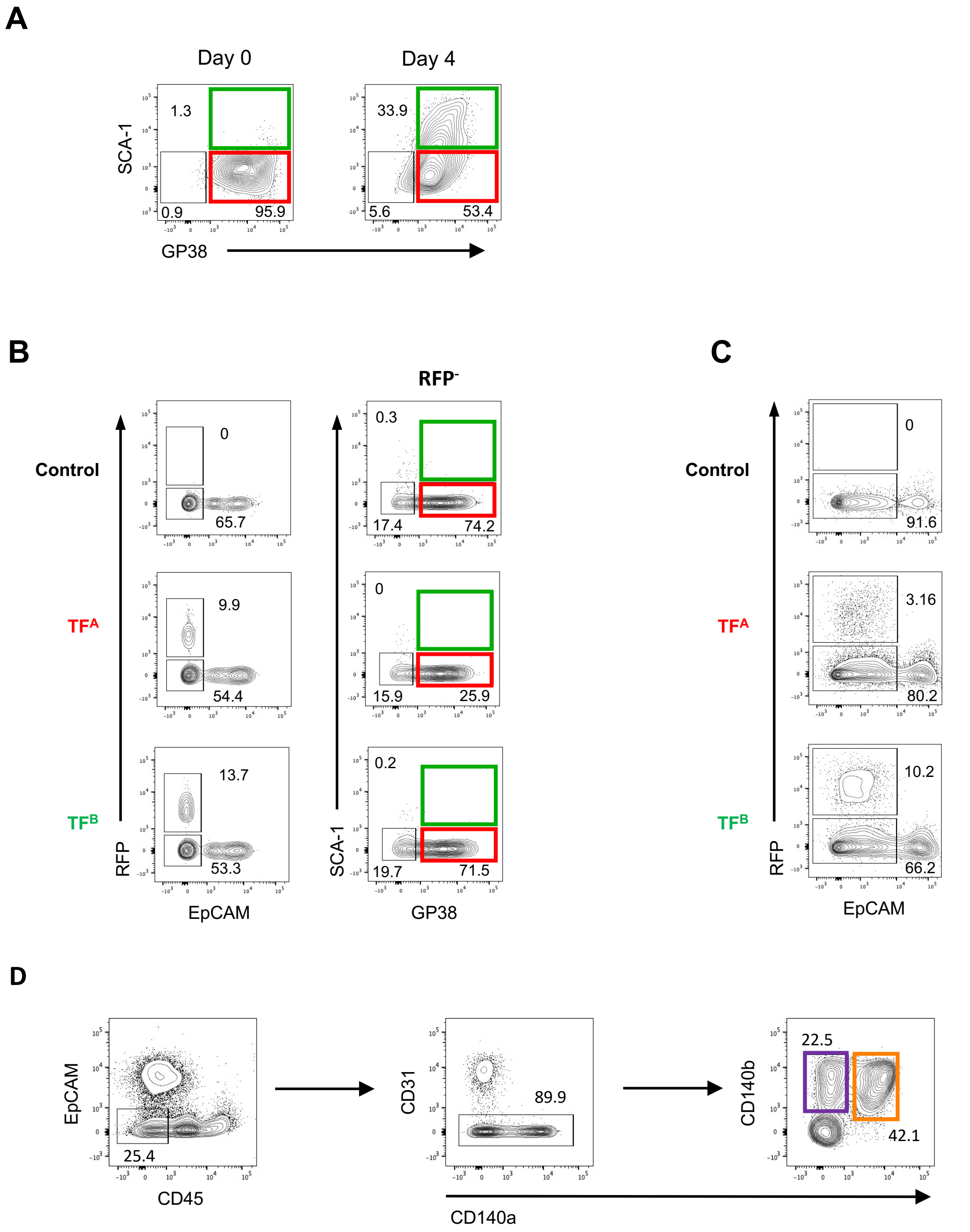

Fig. S3. Precursor-Product relationship between TF subsets. (A) Flow cytometry analysis of the expression pattern of GP38 and SCA-1 at day 0 and after 4 days in culture, from TMCs obtained from fetal thymic organ cultures (FTOC) established with thymic lobes collected from E14 C57BL/6 mice. **(B)** Flow cytometry analysis of day 0 (input) and day 7 (output) RTOC established by combining cells obtained from disaggregated E14 thymus cells from C57BL/6 mice alone (Control) or co-cultured with either TF^A or TF^B cells isolated from Post-natal day P1-P3 Actin-RFP C57BL/6 mice. **(C)** Representative analysis of cells obtained from 1 week-old thymus depicting the gating strategy used to identify TMCs defined as clusters 2 and cluster 3 in *RAG2*$^{-/-}$ *Il2rg*$^{-/-}$. Numbers in plots indicate the frequency of cells found within each gate.

Development • Supplementary information

Table S1. Total normalized counts for all detected genes in the RNAseq analysis of populations TF^A, TF^B and MC.

Click here to download Table S1

Table S2. Total normalized counts and deviation to the mean expression value obtained for genes used as phenotypic markers of populations TF^A, TF^B and MC and for genes previously associated with pericyte and fibroblast cells.

Click here to download Table S2

Table S3. Total normalized counts and deviation to the mean expression value obtained for genes previously associated with capsular fibroblast cells in our TF^A and TF^B cell populations.

Click here to download Table S3

Table S4. Total normalized counts and deviation to the mean expression value obtained for genes previously associated with medullar fibroblast cells in our TF^A and TF^B cell populations.

Click here to download Table S4

Table S5. Total normalized counts of the uniquely upregulated genes of population TF^A in relation to populations TF^B and MC.

Click here to download Table S5

Table S6. Total normalized counts of the uniquely upregulated genes of population TF^B in relation to populations TF^A and MC.

Click here to download Table S6

Table S7. Total normalized counts of the gene ontology analysis of the uniquely upregulated genes of population TF^A.

Click here to download Table S7

Table S8. Total normalized counts of the gene ontology analysis of the uniquely upregulated genes of population TF^B.

Click here to download Table S8

Table S9. Total normalized counts and deviation to the mean expression value obtained for collagen and ECM associated genes in our TF^A and TF^B cell populations.

Click here to download Table S9
