# Supplementary material for: Identification of fibroblast progenitors in the developing mouse thymus
Source: Development. 2022 May 26;149(10):dev200513. doi: 10.1242/dev.200513 (PMC9188757; doi:10.1242/dev.200513)
Supplement: Reviewer comments [file develop-149-200513_review_history.pdf]

Identification of fibroblast progenitors in the developing mouse thymus

Pedro Ferreirinha, Ruben R. G. Pinheiro, Jonathan J. M. Landry and Nuno L. Alves
DOI: 10.1242/dev.200513

Editor: Hanna Mikkola

Review timeline
Original submission: 14 January 2022
Editorial decision: 4 March 2022
First revision received: 14 April 2022
Accepted: 22 April 2022

Original submission

First decision letter

MS ID#: DEVELOP/2022/200513

MS TITLE: Identification of fibroblast progenitors in the developing thymus.

AUTHORS: Pedro Ferreirinha, Ruben RG Pinheiro, Jonathan JM Landry, and Nuno L. Alves

I have now received all the referees' reports on the above manuscript, and have reached a decision. The referees' comments are appended below, or you can access them online: please go to BenchPress and click on the 'Manuscripts with Decisions' queue in the Author Area.

As you will see, the referees express considerable interest in your work, but have some significant criticisms and recommend a substantial revision of your manuscript before we can consider publication. If you are able to revise the manuscript along the lines suggested, which may involve further experiments, I will be happy receive a revised version of the manuscript. Your revised paper will be re-reviewed by one or more of the original referees, and acceptance of your manuscript will depend on your addressing satisfactorily the reviewers' major concerns. Please also note that Development will normally permit only one round of major revision.

We are aware that you may be experiencing disruption to the normal running of your lab that make experimental revisions challenging. If it would be helpful, we encourage you to contact us to discuss your revision in greater detail. Please send us a point-by-point response indicating where you are able to address concerns raised (either experimentally or by changes to the text) and where you will not be able to do so within the normal timeframe of a revision. We will then provide further guidance. Please also note that we are happy to extend revision timeframes as necessary.

Please attend to all of the reviewers' comments and ensure that you clearly highlight all changes made in the revised manuscript. Please avoid using 'Tracked changes' in Word files as these are lost in PDF conversion. I should be grateful if you would also provide a point-by-point response detailing how you have dealt with the points raised by the reviewers in the 'Response to Reviewers' box. If you do not agree with any of their criticisms or suggestions please explain clearly why this is so.

© 2022. Published by The Company of Biologists under the terms of the Creative Commons Attribution License (https://creativecommons.org/licenses/by/4.0/).

Reviewer 1

Advance summary and potential significance to field

The thymus is an organ essential for the development of T cells, the key player of our immune system. Microenvironment of the thymus is comprised of a variety of stromal cells including epithelial cells and fibroblasts. Recently, the role of thymic fibroblasts in thymus organogenesis and T cell development has been highlighted, but their progenitors and developmental pathways remain unclear. In this study, Ferreirinha et al sought to clarify the pathways and mechanisms of thymic fibroblast development. Using multiparameter flow cytometry and transcriptome profiling, the authors identified SCA1- immature fibroblasts in the embryonic thymus and SCA1+ mature fibroblasts comprising the capsule and medulla of the adult thymus. Organ culture experiments with lineage-tracing technique revealed that SCA1- thymic fibroblasts harbor progenitor cell activity. The authors further claimed that the development of thymic fibroblasts depends on signals provided by early developing thymocytes.

This is, I believe, an interesting and timely paper, and will be read by readers in the field of thymus and T-cell biology.

Comments for the author

There are some points that should be addressed to make the authors' conclusion complete and to improve the MS.
#1 Based on the results with Rag2KO/IL2RgKO mice (Fig 3C), the authors concluded that thymic fibroblasts depends on signals provided by early developing thymocytes. However, it is still possibile that IL2Rg (common gamma) in thymic fibroblast themselves is responsible for the phenotype. It seems that IL2Rg and related cytokine receptors are also expressed in thymic stromal cells including fibroblasts, isn't it? If so, it cannot be concluded that the impaired differentiation of thymic fibroblasts in Rag2KO/IL2RgKO mice is due to a defect in thymic crosstalk. This is one of the most important findings in this paper and so should be discussed carefully. The authors should examine the expression levels of IL2Rg and cytokine receptors in thymic stromal cells including fibroblasts. In addition, to directly address whether IL2Rg in thymic fibroblasts is responsible for the phenotype or not, the authors should perform bone marrow chimera experiments in which WT bone marrow cells are transplanted into Rag2KO/IL2RgKO mice and examine whether the maturation of thymic fibroblasts is restored. (Alternatively, the RTOC experiment may be OK, if it works.)
#2 Line 241-252
The method for TMC preparation is not described in detail; even in the Ref. 18, an earlier paper is only cited and no detailed explanation is provided. At least is should be stated in the method section what enzyme(s) were used to dissociate thymic stromal cells (e.g. collagenase, collagenase/dispase, or Liberase). The enzymes used often critically affect the composition of the cells in the cell suspension (Ref. 3), possibly influencing the interpretation of the results.
Also, it should be described how the authors performed intracellular staining for a-SMA.
#3 There is no description about statistics in the method section and figure legends.
#4 The manuscript is not well written. I found some typos (probably more) as follows,
Line 60: 'functionally' -> 'functional'
Line 136-137: 'mural cells (MC) cells' -> delete the second 'cells'
Line 178: 'gave raise to' -> 'gave rise to'
Line 187: 'restricted fibroblast-restricted progenitor activity'
Line 221: 'thymocyte' -> 'thymocytes'
Line 265-266: 'model-based gene set analysis (MGSA) (REF)' -> delete '(REF)' or add reference number

Reviewer 2

Advance summary and potential significance to field

© 2022. Published by The Company of Biologists under the terms of the Creative Commons Attribution License (https://creativecommons.org/licenses/by/4.0/).

The manuscript by Ferreirinha et al. addresses an understudy area of developmental thymus biology, which is the mesenchymal stromal cell compartment. Here the authors elegantly and clearly demonstrate the presence of a thymus-specific fibroblast progenitor, as CD140a+b+GP38+SCA-1- (TFA) cells, and show that they have the capacity to give rise to mature CD140a+b+GP38+SCA-1+ (TFB) thymic fibroblasts. The authors illustrate the developmental progression during thymus ontogeny and take advantage of fetal thymic organ cultures to show that TFA cells give rise to TFB, while TFB cells remain as TFB cells. RNAseq analysis is shown for sorted TFA and TFB cells that clear demonstrates their developmental differences. Lastly, the authors show that progression towards the more mature TFB stage appears to be dependent on lymphocyte-stromal crosstalk, a similar phenomenon to that of thymic epithelial cell maturation. The authors also reflect on how their findings align with the recently described thymic fibroblast subsets, capsular DDP4+ and medullary DDP4- cells; but fall short in showing whether each of these subsets contains a unique of shared progenitor within the TFA subset, which appears to contain both DPP4- and DPP4+ cells. Nevertheless, the work is highly timely and reveals new insights as to how different stromal cells differentiate and are maintained in the adult thymus.

Comments for the author

The manuscript by Ferreirinha et al. addresses an understudy area of developmental thymus biology, which is the mesenchymal stromal cell compartment. Here the authors elegantly and clearly demonstrate the presence of a thymus-specific fibroblast progenitor, as CD140a+b+GP38+SCA-1- (TFA) cells, and show that they have the capacity to give rise to mature CD140a+b+GP38+SCA-1+ (TFB) thymic fibroblasts. The authors illustrate the developmental progression during thymus ontogeny and take advantage of fetal thymic organ cultures to show that TFA cells give rise to TFB, while TFB cells remain as TFB cells. RNAseq analysis is shown for sorted TFA and TFB cells that clear demonstrates their developmental differences. Lastly, the authors show that progression towards the more mature TFB stage appears to be dependent on lymphocyte-stromal crosstalk, a similar phenomenon to that of thymic epithelial cell maturation. The authors also reflect on how their findings align with the recently described thymic fibroblast subsets, capsular DDP4+ and medullary DDP4- cells; but fall short in showing whether each of these subsets contains a unique of shared progenitor within the TFA subset, which appears to contain both DPP4- and DPP4+ cells. Nevertheless, the work is highly timely and reveals new insights as to how different stromal cells differentiate and are maintained in the adult thymus.

1- As pointed out above, if possible, it would be good to show whether the TFA cells seen in the 1W thymus (and earlier, Figure 1C) that are DPP4- and DPP4+ are able to uniquely give rise to DPP4- and DPP4+, respectively, when transferred into a host FTOC as done in Figure 3; or whether the DPP4+ TFA cell give rise to both DPP4- and DPP4+ cells, and vice versa for DPP4- TFA cell.

2- One question that can be readily addressed (of note: it was difficult to evaluate this question as this reviewer could not access the supplemental tables due to a file error), is whether the TFA or TFB cells show differential expression for LTbR (and other TNFRs), since this signaling pathway, as pointed out by the authors, was recently shown to be critical for the function of medullary fibroblasts. This analysis can be added to Figure 2 heat map, for members of the TNFR gene family.

First revision

Author response to reviewers' comments

We are pleased that the reviewers recognized the scientific quality of the study. We thank them for their useful comments. Within the allowed time limit, we addressed all their comments in the point-by-point reply and revised manuscript (the comments by the reviewers are transcribed *in italic type*).

Reviewer 1: *"The thymus is an organ essential for the development of T cells, the key player of our immune system. Microenvironment of the thymus is comprised of a variety of stromal cells including epithelial cells and fibroblasts. Recently, the role of thymic fibroblasts in thymus*

© 2022. Published by The Company of Biologists under the terms of the Creative Commons Attribution License (https://creativecommons.org/licenses/by/4.0/).

organogenesis and T cell development has been highlighted, but their progenitors and developmental pathways remain unclear. In this study, Ferreirinha et al sought to clarify the pathways and mechanisms of thymic fibroblast development. Using multiparameter flow cytometry and transcriptome profiling, the authors identified SCA1- immature fibroblasts in the embryonic thymus and SCA1+ mature fibroblasts comprising the capsule and medulla of the adult thymus. Organ culture experiments with lineage-tracing technique revealed that SCA1- thymic fibroblasts harbor progenitor cell activity. The authors further claimed that the development of thymic fibroblasts depends on signals provided by early developing thymocytes. This is, I believe, an interesting and timely paper, and will be read by readers in the field of thymus and T- cell biology."

REPLY: We thank the reviewer for the overall positive appreciation of our study and insightful notes.

#1 Based on the results with Rag2KO/IL2RgKO mice (Fig 3C), the authors concluded that thymic fibroblasts depends on signals provided by early developing thymocytes. However, it is still possibile that IL2Rg (common gamma) in thymic fibroblast themselves is responsible for the phenotype. It seems that IL2Rg and related cytokine receptors are also expressed in thymic stromal cells including fibroblasts, isn't it? If so, it cannot be concluded that the impaired differentiation of thymic fibroblasts in Rag2KO/IL2RgKO mice is due to a defect in thymic crosstalk. This is one of the most important findings in this paper and so should be discussed carefully. The authors should examine the expression levels of IL2Rg and cytokine receptors in thymic stromal cells including fibroblasts. In addition, to directly address whether IL2Rg in thymic fibroblasts is responsible for the phenotype or not, the authors should perform bone marrow chimera experiments in which WT bone marrow cells are transplanted into Rag2KO/IL2RgKO mice and examine whether the maturation of thymic fibroblasts is restored. (Alternatively, the RTOC experiment may be OK, if it works.)

REPLY: We agree with the reviewer that these results represent important findings in our study. We would like to respectfully point out that fibroblast differentiation was also affected in *Rag2*$^{-/-}$ mice, wherein γ_c- mediated signalling was intact. Although the frequency of TF^B in the 4-week-old *Rag2*$^{-/-}$ thymus matched the one found in WT thymus, the proportion of this subset was reduced in the 1-week-old *Rag2*$^{-/-}$ thymus. The effects in the frequency of TF^B on *Rag2*$^{-/-}$ was less prominent as compared to *Rag2*$^{-/-}$*Il2rg*$^{-/-}$. Still, the cellularity of TF^B subset was severely affected in both *Rag2*$^{-/-}$ and *Rag2*$^{-/-}$*Il2rg*$^{-/-}$. These results suggest that stromal interactions with thymocytes passing ß selection promote the regular differentiation program of thymic fibroblasts. We have revised the description of these results and the representation of data in Fig.3. We agree that we cannot formally exclude an additional involvement for IL2Rγ-mediated signalling in thymic fibroblasts. Mutations in γ_c cytokines and their receptor chains are mostly known to affect hematopoietic cells[2]. There are very few studies reporting the role of γ_c cytokines in non-hematopoietic cells. IL-2R subunits are expressed in endothelial cells and IL-2 regulates their permeability. IL-4 seems to act on neurons to promote the axonal repair [2]. We are not aware of reports demonstrating the effect of γ_c-mediated signalling in mesenchymal cells. In our study, we were not able to measure the protein levels of all γ_c receptor chains, as we did not have immediate access to Abs within the timeframe of this submission. We analysed our RNA seq data and observed that *Il2rg* was not differentially expressed in TF^A and TF^B subsets. Moreover, the expression of γ_c receptor chains was very low or hardly detected, when compared to other receptors (and markers) typically expressed in fibroblast (**Fig. 1A**, for the reviewers only). The counts for all genes detected in TF subsets were included in Table S1. As we did not include a bona-fide γ_c expressing population (ex. T cells), we seek available transcriptomic data wherein hematopoietic and mesenchymal cells were co-analysed [1]. *Il2rg* expression was expectedly downregulated in thymic mesenchymal cells as compared to thymocytes (**Fig. 1B**). Although these data represented an assessment at the mRNA level, they suggest a low expression of *Il2rg* and co-receptors for γ_c cytokines in mesenchymal cells.

© 2022. Published by The Company of Biologists under the terms of the Creative Commons Attribution License (https://creativecommons.org/licenses/by/4.0/).

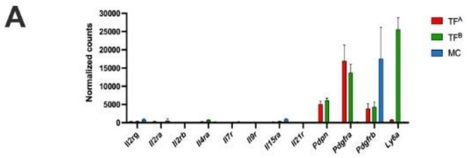

NOTE: We have removed unpublished data that had been provided for the referees in confidence.

Figure 1 - A) Normalized counts for the expression of γ_c cytokine receptor chains in thymic fibroblast subsets and vascular- associated mural cells. **B)** Expression of *Il2rg* is represented as the difference in the expression of the gene found in thymocytes (Thy), thymic mesenchymal (TMC), endothelial (EC) and epithelial (TEC) cells relatively to the average expression as in[1].

The experiments suggested by the reviewer (RTOC/BM transplantation) are interesting. However, we have had limited access to *Rag2*$^{-/-}$ and *Rag2*$^{-/-}$*Il2rg*$^{-/-}$ mice and have not been able to secure enough aged- matched animals to conduct them during the timeframe provided for this revision. Although we cannot formally exclude an additional role for γ_c signalling in thymic fibroblast or stromal cells, we consider that our data in *Rag2*$^{-/-}$ and *Rag2*$^{-/-}$*Il2rg*$^{-/-}$ thymus collectively suggest that cooperative signals from thymocytes passing ß-selection are important to ensure the normal maturation of thymic fibroblasts. We have expanded (within the size limit) the discussion on this point, attenuating the strength of some conclusions (pgs 8-9, lines 208-223, 246-248).

#2 Line 241-252 The method for TMC preparation is not described in detail; even in the Ref. 18, an earlier paper is only cited and no detailed explanation is provided. At least is should be stated in the method section what enzyme(s) were used to dissociate thymic stromal cells (e.g. collagenase, collagenase/dispase, or Liberase). The enzymes used often critically affect the composition of the cells in the cell suspension (Ref. 3), possibly influencing the interpretation of the results.
Also, it should be described how the authors performed intracellular staining for a-SMA.
REPLY: We thank the reviewer for these notes and we apologize for the non-deliberate omission. We have edited our text in material and methods (pg 9 lines 242-249 and 260-263).

#3 There is no description about statistics in the method section and figure legends.
REPLY: We thank the reviewer for these notes and we apologize for the non-deliberate omission. We have edited our text in material and methods (pgs 9-10 lines 276-287), figures and legends.

#4 The manuscript is not well written. I found some typos (probably more) as follows, Line 60: 'functionally' -> 'functional'
Line 136-137: 'mural cells (MC) cells' -> delete the second 'cells Line 178: 'gave raise to' -> 'gave rise to'
Line 187: 'restricted fibroblast-restricted progenitor activity' Line 221: 'thymocyte' -> 'thymocytes'
Line 265-266: 'model-based gene set analysis (MGSA) (REF)' -> delete '(REF)' or add reference number
REPLY: We thank the reviewer for these notes. We have thoroughly revised the text and corrected this and additional typos and incongruences found in the manuscript.

Reviewer 2: *The manuscript by Ferreirinha et al. addresses an understudy area of developmental thymus biology, which is the mesenchymal stromal cell compartment. Here the authors elegantly and clearly demonstrate the presence of a thymus-specific fibroblast progenitor, as CD140a+b+GP38+SCA-1- (TFA) cells, and show that they have the capacity to give rise to mature CD140a+b+GP38+SCA-1+ (TFB) thymic fibroblasts. The authors illustrate the developmental progression during thymus ontogeny and take advantage of fetal thymic organ cultures to show that TFA cells give rise to TFB, while TFB cells remain as TFB cells. RNAseq analysis is shown for sorted TFA and TFB cells that clear demonstrates their developmental differences. Lastly, the authors show that progression towards the more mature TFB stage appears to be dependent on lymphocyte-stromal crosstalk, a similar phenomenon to that of thymic epithelial cell maturation. The authors also reflect on how their findings align with the recently described*

© 2022. Published by The Company of Biologists under the terms of the Creative Commons Attribution License (https://creativecommons.org/licenses/by/4.0/).

thymic fibroblast subsets, capsular DDP4+ and medullary DDP4- cells; but fall short in showing whether each of these subsets contains a unique of shared progenitor within the TFA subset, which appears to contain both DPP4- and DPP4+ cells. Nevertheless, the work is highly timely and reveals new insights as to how different stromal cells differentiate and are maintained in the adult thymus.

REPLY: We thank the reviewer for the positive evaluation of our study and insightful comments.

1- As pointed out above, if possible, it would be good to show whether the TFA cells seen in the 1W thymus (and earlier, Figure 1C) that are DPP4- and DPP4+ are able to uniquely give rise to DPP4- and DPP4+, respectively, when transferred into a host FTOC as done in Figure 3; or whether the DPP4+ TFA cell give rise to both DPP4- and DPP4+ cells, and vice versa for DPP4- TFA cell.

REPLY: We thank the reviewer for this note. The experiments suggested by the reviewer are interesting and relate to the lineage relationship between capsular and medullary fibroblasts. We would like to respectfully call the attention of the reviewer to the following aspects:

(I) At E14.5, a time wherein TF^B were virtually absent, $DPP4^+$ and $DPP4^-$ cells were already detected within TF^A. Nitta and colleagues [3] also reported that the first $DPP4^+$ appear around E14.5-15.5, a period concordant with our observations.

(II) The primordial TF^B ($SCA\text{-}1^+$ cells) appeared at E17 and were mostly defined as $DPP4^+$. Their developmental trajectory suggested that they may arise from precursors residing within $DPP4^+$ TF^A.

(III) From the postnatal period onwards, TF^B contained both $DPP4^-$ and $DPP4^+$ cells, and thus it is unclear whether TF^B $DPP4^-$ may arise from TF^B $DPP4^+$ cells and/or from $DDP4^-$ TF^A(**Fig. 1D**).

Our data indicate that SCA-1 is acquired firstly by capsular ($DDP4^+$) fibroblast followed by medullary ($DDP4^-$) counterparts, and as such, did not by itself discriminate these subsets. Thus, Sca1 appears to represent a maturation marker commonly acquired in $DDP4^-$ and $DDP4^+$ thymic fibroblasts. Concordantly, we found that capsular- and medullary-associated genes were both upregulated in TF^B(**FigS1B).**

Our result and the one's by Nitta and colleagues [3] suggested that the segregation of capsular and medullary subtypes may occur early in thymic development. Yet, as the reviewer indicates they do not exclude the possibility that capsular give rise to medullary subsets, and vice-versa. Although this point was also not originally explored in the study by Nitta *et al.*, it was not the primary scope of our study, as we found that Sca1 is a marker commonly expressed by both capsular and medullary subsets. We agree with the reviewer that several developmental trajectories can be hypothesized for $DPP4^+$ and $DPP4^-$ found within TF^A, but also TF^B. Yet, we consider that these analyses would require complementary and complex analysis that will extend beyond the time frame of our side. Particularly, we consider that it would be important to determine the lineage potential of $DDP4^-$ and $DDP4^+$ subsets within TF^A or TF^B at different embryonic and postnatal stages, as they may (or not) harbour distinct uni- or bi-potent precursors that differ during development. Moreover, these analyses should also extend to the potential of TF subsets to convert to other mesenchymal cells, such as pericytes, as it has been shown for $CD34^+$ adventitial cells [4]. Although the proposed experiments are "virtually" possible, they would also require a substantial number of mice, given that thymic fibroblasts are an extremely rare subset. Moreover, they would require an additional complementary microscopy analysis to define the location of these putative precursors and their progeny (capsule vs medulla). During the time of the revision, we attempted to establish RTOCs with embryonic E14 DPP4- and DPP4+ TF^A but we failed to purify enough cells to find their progeny following culture. Thus, the implementation of the complete experiments would require scaling up tremendously the number of C57Bl/6 WT (RTOC carriers cells) and $Actin^{RFP}$ (source of spiked TF), which is virtually impossible in the time frame of this resubmission. We reason that the studies are worth pursuing, but may extend beyond the timeframe of this resubmission. We covered the aforementioned points in the revised version (pg 6, lines 128-139 and pg9 lines 225-233, 246-248).

2- One question that can be readily addressed (of note: it was difficult to evaluate this question as this reviewer could not access the supplemental tables due to a file error), is whether the TFA or

© 2022. Published by The Company of Biologists under the terms of the Creative Commons Attribution License (https://creativecommons.org/licenses/by/4.0/).

TFB cells show differential expression for LTbR (and other TNFRs), since this signaling pathway, as pointed out by the authors, was recently shown to be critical for the function of medullary fibroblasts.

REPLY: We thank the reviewer for this note. We have analysed all members of the TNFRSF family and found that *Ltbr, Tnfrsf1b, Tnfrsf12a* and *Tnfrsf23* were specifically upregulated in TF^B (**Fig S2E and TableS1 and 6**). We included this analysis in the revised version (pg 7, lines 173-176; pg 9 lines 246-248). We are sorry that the reviewer had problems in visualizing the supplementary tables. As far as we could confirm during our submission, all supplemental tables were uploaded as a single pdf file. We confirmed that they were ok before submitting the manuscript. We uploaded new versions of the tables to facilitate their analysis in the revised version.

References

Kernfeld, E. M. et al. A Single-Cell Transcriptomic Atlas of Thymus Organogenesis Resolves Cell Types and Developmental Maturation. Immunity 48, 1258-1270 e1256, doi:10.1016/j.immuni.2018.04.015 (2018).
Leonard, W. J., Lin, J. X. & O'Shea, J. J. The gammac Family of Cytokines: Basic Biology to Therapeutic Ramifications. Immunity 50, 832-850, doi:10.1016/j.immuni.2019.03.028 (2019).
Nitta, T. et al. Fibroblasts as a source of self-antigens for central immune tolerance. Nat Immunol 21, 1172-1180, doi:10.1038/s41590-020-0756-8 (2020).
Sitnik, K. M. et al. Context-Dependent Development of Lymphoid Stroma from Adult CD34(+) Adventitial Progenitors. Cell Rep 14, 2375-2388, doi:10.1016/j.celrep.2016.02.033 (2016).

Second decision letter

MS ID#: DEVELOP/2022/200513

MS TITLE: Identification of fibroblast progenitors in the developing thymus.

AUTHORS: Pedro Ferreirinha, Ruben RG Pinheiro, Jonathan JM Landry, and Nuno L. Alves
ARTICLE TYPE: Research Report

I am happy to tell you that your manuscript has been accepted for publication in Development, pending our standard ethics checks.

Reviewer 1

Advance summary and potential significance to field

The thymus is an organ essential for the development of T cells, the key player of our immune system. Microenvironment of the thymus is comprised of a variety of stromal cells including epithelial cells and fibroblasts. Recently, the role of thymic fibroblasts in thymus organogenesis and T cell development has been highlighted, but their progenitors and developmental pathways remain unclear. In this study, Ferreirinha et al sought to clarify the pathways and mechanisms of thymic fibroblast development. Using multiparameter flow cytometry and transcriptome profiling, the authors identified SCA1- immature fibroblasts in the embryonic thymus and SCA1+ mature fibroblasts comprising the capsule and medulla of the adult thymus. Organ culture experiments with lineage-tracing technique revealed that SCA1- thymic fibroblasts harbor progenitor cell activity. The authors further claimed that the development of thymic fibroblasts depends on signals provided by early developing thymocytes.

This is, I believe, an interesting and timely paper, and will be read by readers in the field of thymus and T-cell biology.

Comments for the author

#1 My point has been properly discussed in the Discussion section.

© 2022. Published by The Company of Biologists under the terms of the Creative Commons Attribution License (https://creativecommons.org/licenses/by/4.0/).

#2 The method for TMC preparation is appropriately described.

Reviewer 2

Advance summary and potential significance to field

The manuscript by Ferreirinha et al. addresses an understudy area of developmental thymus biology, which is the mesenchymal stromal cell compartment. Here the authors elegantly and clearly demonstrate the presence

of a thymus-specific fibroblast progenitor, as CD140a+b+GP38+SCA-1- (TFA) cells, and show that they have the capacity to give rise to mature CD140a+b+GP38+SCA-1+ (TFB) thymic fibroblasts. The authors illustrate the developmental progression during thymus ontogeny and take advantage of fetal thymic organ cultures to show that TFA cells give rise to TFB, while TFB cells remain as TFB cells. RNAseq analysis is shown for sorted

TFA and TFB cells that clear demonstrates their developmental differences. Lastly, the authors show that progression towards the more mature TFB stage appears to be dependent on lymphocyte-stromal crosstalk, a similar phenomenon to that of thymic epithelial cell maturation. The authors also reflect on how their findings align with the recently described thymic fibroblast subsets, capsular DDP4+ and medullary DDP4- cells; but fall short in showing whether each of these subsets contains a unique of shared progenitor within the TFA subset, which appears to contain both DPP4- and DPP4+ cells. Nevertheless, the work is highly timely and reveals new insights as to how different stromal cells differentiate and are maintained in the adult thymus.

Comments for the author

The authors provide a fairly reasonable argument as to why the lineage precursor product analysis of TFA subsets based on DDP4 expression, while worthwhile, it appears to be a challenging task and can be part of a follow up paper. Nonetheless, the authors have added this point to the discussion, and provided a thoughtful set of potential lineage outcomes based on their findings and those in the literature.

© 2022. Published by The Company of Biologists under the terms of the Creative Commons Attribution License (https://creativecommons.org/licenses/by/4.0/).
